# Does Compassion Predict Blood Pressure and Hypertension? The Modifying Role of Familial Risk for Hypertension

**DOI:** 10.1007/s12529-020-09886-5

**Published:** 2020-04-28

**Authors:** Aino I. L. Saarinen, Liisa Keltikangas-Järvinen, Taina Hintsa, Laura Pulkki-Råback, Niklas Ravaja, Terho Lehtimäki, Olli Raitakari, Mirka Hintsanen

**Affiliations:** 1 Research Unit of Psychology, University of Oulu, P.O. Box 2000 (Erkki Koiso-Kanttilan katu 1), 90014 Oulu, Finland; 2 Department of Psychology and Logopedics, Faculty of Medicine, University of Helsinki, Helsinki, Finland; 3 Department of Educational Sciences and Psychology, University of Eastern Finland, Joensuu, Finland; 4 Department of Clinical Chemistry, Fimlab Laboratories and Finnish Cardiovascular Research Center-Tampere, Faculty of Medicine and Health Technology, Tampere University, Tampere, Finland; 5 Research Centre of Applied and Preventive Cardiovascular Medicine, University of Turku, Turku, Finland; 6 Department of Clinical Physiology and Nuclear Medicine, Turku University Hospital, Turku, Finland; 7 Centre for Population Health Research, University of Turku and Turku University Hospital, Turku, Finland

**Keywords:** Blood pressure, Compassion, Personality, Familial risk, Health behavior, Hypertension

## Abstract

**Background:**

This study investigated (i) whether compassion is associated with blood pressure or hypertension in adulthood and (ii) whether familial risk for hypertension modifies these associations.

**Method:**

The participants (*N* = 1112–1293) came from the prospective Young Finns Study. Parental hypertension was assessed in 1983–2007; participants’ blood pressure in 2001, 2007, and 2011; hypertension in 2007 and 2011 (participants were aged 30–49 years in 2007–2011); and compassion in 2001.

**Results:**

High compassion predicted lower levels of diastolic and systolic blood pressure in adulthood. Additionally, high compassion was related to lower risk for hypertension in adulthood among individuals with no familial risk for hypertension (independently of age, sex, participants’ and their parents’ socioeconomic factors, and participants’ health behaviors). Compassion was not related to hypertension in adulthood among individuals with familial risk for hypertension.

**Conclusion:**

High compassion predicts lower diastolic and systolic blood pressure in adulthood. Moreover, high compassion may protect against hypertension among individuals without familial risk for hypertension. As our sample consisted of comparatively young participants, our findings provide novel implications for especially early-onset hypertension.

**Electronic supplementary material:**

The online version of this article (10.1007/s12529-020-09886-5) contains supplementary material, which is available to authorized users.

## Introduction

The World Health Organization (WHO) has estimated that raised blood pressure causes even 12.8% of all deaths [1]. There is a great amount of evidence that even modest decreases in systolic blood pressure predict a substantially lower risk for major cardiovascular disease events, coronary heart disease, stroke, and heart failure [2, 3]. By now, it is widely known that certain psychological traits, especially anxiety and depression, increase the risk for raised blood pressure [4, 5]. However, recent research suggests that focusing on the positive determinants of health—instead of the negative ones—may be more helpful in obtaining the American Heart Association’s health goals of improving cardiovascular health [6]. Although some research suggests that personalities with higher emotional vitality have better cardiovascular health [7], the current evidence has remained scarce with regard to which specific positive personality traits that might promote better cardiovascular health and protect against raised blood pressure and hypertension.

Dispositional compassion for others might be one essential trait protecting against adverse cardiovascular outcomes. Compassion is defined as a disposition to feel concern for other’s suffering that is followed by the desire to alleviate the suffering [8]. Dispositional compassion includes three characteristics that may make it especially important in the context of heart diseases. Firstly, the onset of heart diseases is known to be affected both by affective and behavioral factors. Contrary to most previously investigated psychosocial factors (e.g., positive affect or amount of exercise), compassion includes simultaneously both an affective element (recognizing close other’s emotional distress and tolerating it) and a behavioral element (willingness to take the behavioral steps to reduce other’s distress) [9, 10]. Hence, compassion might include a beneficial psychological profile in the light of heart diseases. Secondly, the benefits of most psychosocial qualities are context-dependent: for example, the effectiveness of specific stress-coping strategies is very dependent on the psychosocial context where they take place [11]. Instead, compassion is suggested to represent a fundamental need for human being since it promotes possibilities to live in harmony with important others and protects from social exclusion [12]. Finally, while positive affectivity and other temperament traits have quite a strong biological basis, compassion is susceptible for environmental factors and can be effectively trained even within a relatively short time period [13–16], providing practical implications for interventions.

Additionally, personality traits may promote better cardiovascular health via more frequent favorable health behaviors [17, 18]. It has been demonstrated that high compassion is linked with a lower risk for excessive energy intake and eating-related problem behavior [19], with a lower risk for at-risk alcohol use and smoking [20, 21], and with lower consumption of some caffeine-including drinks [22]. Modification of those health behaviors, in turn, is shown to be a very effective treatment for raised blood pressure [23, 24]. Taken together, high compassion may likely promote favorable health behaviors and, in that way, protect against the incidence of raised blood pressure and hypertension.

Importantly, in addition to health behaviors, also genetic factors have a substantial influence on the incidence of raised blood pressure [25]. There is also evidence for a substantial transmission of hypertension from parents to their offspring [26]. Overall, it has been estimated that genetic factors contribute to as much as 50% of blood pressure variation [27].

The interaction between genetic factors and psychosocial factors in the occurrence of cardiovascular diseases has been widely demonstrated [27]. For those with familial risk for hypertension, the causes lie to larger extent in genes whereas health behaviors play somewhat a lesser role [28]. Previously, even such statements have been proposed that some individuals may have inherited hypertensive levels of blood pressure [28]. Accordingly, the relationship of compassion with blood pressure may be different among individuals with and without a familial risk for hypertension. Specifically, the potential protective role of compassion against the development of hypertension may be limited among individuals with a strong genetic risk for hypertension. Consequently, compassion may potentially protect against hypertension more strongly among such individuals who do not have familial risk for hypertension and whose hypertension is more strongly linked to adverse health behaviors, such as smoking, alcohol use, and obesity. This topic, however, has still remained unexplored.

To our knowledge, this is the first study to investigate the relationship of compassion for others with blood pressure and hypertension. Specifically, the aim of the present study was to examine (i) whether compassion for others is associated with diastolic or systolic blood pressure or hypertension in adulthood and (ii) whether a familial risk for hypertension modifies the association of compassion with hypertension and blood pressure. Intergenerational data with a 31-year prospective follow-up was used. The data provided possibilities to control for a wide range of covariates, including age, sex, participants’ and their parents’ socioeconomic factors, smoking status, alcohol use, body mass index, physical activity, and coffee consumption.

## Materials and Methods

### Participants

We used data from the prospective Young Finns Study. The participants were selected randomly from six age cohorts (born between 1962 and 1977) from the population register of the Social Insurance Institution. The Social Insurance Institution covers the whole population of Finland. The original sample included 3596 participants in the baseline measurement in 1980 (when participants were aged 3–18 years). The participants have been followed since then so that the latest follow-up measurement was in 2011 (participants were aged 24–49 years). The study was carried out in accordance with the Declaration of Helsinki. Furthermore, the design of the Young Finns Study was approved by all the Finnish universities with medical schools. Before participation, all the participants or their parents (for participants aged below 12 years) provided informed consent after the nature of the procedures had been fully explained. The design of the Young Finns Study is described with more detail elsewhere [29].

For this study, parental hypertension was assessed in 1980, 1983, 1986, 1989, 1992, 2001, and 2007; offspring’s blood pressure and the use of antihypertensive medications in 2001, 2007, and 2011; offspring’s hypertension in 2007 and 2011; compassion in 2001; parental socioeconomic factors in 1980; offspring’s socioeconomic factors in 2011; body mass index, smoking status, alcohol use, and physical activity in 2001, 2007, and 2011; and coffee consumption in 2001. A timeline of the study design is available in Supplementary Table 1. In the analyses, all the participants with data on study variables in at least one of the measurement times (e.g., data available on diastolic blood pressure in 2001, 2007, or 2011; or on offspring’s hypertension in 2007 or 2011) were included. Hence, the sample size slightly varied between the analyses (1112–1293 participants).

### Measures

#### Dispositional Compassion

Dispositional compassion was evaluated with the version 9 of the Temperament and Character Inventory [30]. Dispositional compassion is a subscale of the character dimension Cooperativeness of the TCI. The scale of dispositional compassion (*α* = .86 in the sample of this study) consists of 10 self-rating statements (e.g., “It gives me pleasure to help others, even if they have treated me badly”; “It gives me pleasure to see my enemies suffer” [reverse scored]) that are rated with a 5-point Likert scale ranging from 1 (completely disagree) to 5 (completely agree). The mean score of compassion was calculated for all the participants who had data on at least 50% of the items. In the analyses, the standardized score (mean = 0; SD = 1; 10th percentile = − 1.293; 90th percentile = 1.094) of dispositional compassion was used. Previous studies have confirmed the reliability of the scale [31]. Moreover, the convergent and discriminant validity has also been confirmed, described in detail elsewhere [32]. Specifically, high values of the dispositional compassion have been shown to correlate with higher sociability, altruistic behavior, and positive emotionality [33, 34] whereas low values of the compassion are related to higher hostility, aggression, and narcissistic and psychopathic features [33, 35–37].

#### Diastolic and Systolic Blood Pressure and Antihypertensive Medications

Blood pressure was measured in sitting position after 5-min rest. A mercury sphygmomanometer at phases 1 and 2 and with a random zero sphygmomanometer (Hawksley & Sons Ltd) at phase 3 was used. Cuff size for the measurement covered two-thirds of the participant’s arm length. Korotkoff’s first phase was determined as the indicator of systolic blood pressure. Readings to the nearest even number of millimeters of mercury were conducted 3 times for each participant. In the analyses, the average values of diastolic and systolic blood pressure were used between the three measurements. Information about the use of antihypertensive was derived from health care records. Use of antihypertensive medications was included as covariate in the analyses when predicting blood pressure.

#### Hypertension

The presence of participants’ and their parents’ hypertension was evaluated using self-rating questionnaires. Participants and their parents were asked whether they had been diagnosed with hypertension. A two-class variable was computed referring to whether participants had been diagnosed with hypertension or not in 2007 or 2011 (0 = not diagnosed; 1 = diagnosed). Regarding the familial risk for hypertension, a variable was computed indicating whether at least one parent reported having been diagnosed with hypertension in 1980, 1983, 1986, 1989, 1992, 2001, or 2007 (0 = not diagnosed; 1 = diagnosed).

When defining indicators of hypertension, the current study did not use cut-off scores of systolic and diastolic blood pressure or the use of antihypertensive medications because this would have resulted in ambiguities and potential for bias. That is, the cut-off points for blood pressure have remained a controversial topic (various cut-offs have been suggested) and, additionally, different cut-offs have been recommended for subpopulations like diabetics [38]. Furthermore, repeated measurements in various environments (that were not available) are needed before diagnosing hypertension [38]. Antihypertensive medications are often used for a variety of purposes (e.g., for migraine) in Finland (see the Finnish current care guidelines here: https://www.kaypahoito.fi/en/) and are, thus, not necessarily a reliable indicator of hypertension. Also in our sample, there were participants who reported having been diagnosed by a doctor with migraine, cerebral infarction, or psychiatric disorders (but not with hypertension) and who used antihypertensive medications. Hence, many participants seemed to be using antihypertensive medications due to other somatic or neuropsychiatric diseases.

#### Socioeconomic Factors

Participants’ and their parents’ socioeconomic factors included education and level of income. Parental educational level was classified into 3 categories (1, comprehensive school; 2, high school or occupational school; 3, academic level). Level of parental income included 8 categories (1, less than 15,000 Finnish mark (2523€) per year; 8, more than 100,000 Finnish mark (16,819€) per year). Participants’ education was defined as the number of educational years (ranging between 8 and 30 years). Participants’ level of income was assessed with a 13-point scale (1 = less than 5000€ per year; 13 = more than 60,000€ per year). All the continuous socioeconomic variables (i.e., parents’ level of income, participants’ number of educational years, participants’ level of income) were standardized (mean = 0, SD = 1). The socioeconomic factors were added to the analyses as separate variables.

#### Health Behaviors

Covariates included coffee consumption, smoking status, alcohol use, body mass index, physical activity, and socioeconomic factors in childhood and adulthood. Coffee consumption was defined as the number of cups of coffee per day. Body mass index was calculated as weight (kg) divided by height squared (m^2^). Body mass index was included in health behaviors since it reflects the balance between energy intake and consumption. Smoking status was determined by asking the participants how often they were smoking (1 = daily smoking; 5 = never smoked). Smoking status was classified into two categories (1 = daily smoking; 0 = not daily smoking). Alcohol use was measured by asking the participants for the number of intoxications per year (i.e., the use of 6 or more portions of alcohol at a time). The scale ranged from 1 (2 times or more per week) to 6 (less than once a year). All the covariates were standardized with the mean of 0 and standard deviation of 1.

The scale of physical activity included 5 items asking about participants’ physical activity (items can be found in Supplementary Material). The total score of physical activity was defined as the standardized mean of the standardized items (mean = 0, SD = 1), so that each item had an equal weight for the total score of physical activity. The mean score of physical activity was calculated for all the participants who had data on at least 50% of the items. The internal consistency for the scale of physical activity ranged between *α* = .80 and .83 in 2001–2011. This scale of physical activity has been used also previously [39].

### Statistical Analyses

Attrition was examined by comparing the included and excluded participants with regard to study variables using chi-square tests and independent samples *t* tests. The association of offspring’s compassion with diastolic and systolic blood pressure was investigated using linear regression analyses. The mean scores of diastolic and systolic blood pressure between years 2007 and 2011 were predicted by compassion. The mean scores of diastolic and systolic blood pressure were calculated for all the participants with data available on blood pressure in baseline measurement 2001 and also in at least one of the outcome measurement points (2007 and 2011) (*N* = 1103). Model 1 was adjusted for diastolic/systolic blood pressure at the baseline measurement in 2001, age, sex, and use of antihypertensive medications in 2001, 2007, and 2011; model 2 was adjusted also for participants’ and their parents’ socioeconomic factors; and model 3 was adjusted also for health behaviors (coffee consumption, smoking status, alcohol use, body mass index, and physical activity).

Next, the associations of familial risk for hypertension and offspring’s compassion with hypertension were investigated using logistic regression analyses where offspring’s hypertension was predicted by compassion, familial risk for hypertension, and their interaction. Model 1 was adjusted for age and sex; model 2 also for participants’ and their parents’ socioeconomic factors; and model 3 also for health behaviors (smoking status, alcohol use, body mass index, physical activity, and coffee consumption). In the logistic regression analyses, the standardized mean scores of indicators of health behaviors (smoking status, alcohol use, body mass index, physical activity) between measurement years 2001, 2007, and 2011 were used. The mean scores of health behaviors were calculated for all the participants who had data available about health behaviors in at least one of the measurement years.

## Results

The descriptive statistics of the study variables are shown in Table 1. Attrition analyses revealed that women were more likely to participate than men (41.3% vs. 31.3%, *p* < .001). Included participants were slightly older than excluded participants (31.67 vs. 31.31, *p* < .05). There was no attrition bias in the level of compassion or in the frequency of hypertension. However, included participants’ parents had more likely hypertension than excluded participants’ parents (63.2% vs. 52.3%, *p* < .001). Additionally, included participants had slightly lower level of diastolic blood pressure (74.87 vs. 76.20, *p* < .01) and systolic blood pressure (119.17 vs. 121.15, *p* < .001) than excluded participants. With regard to health behaviors, it was found that included participants had lower level of coffee consumption (3.25 vs. 3.68, *p* < .001), were less likely to smoke daily (17.6% vs. 23.8%, *p* < .001), used alcohol less frequently (4.57 vs. 4.31, *p* < .001), and were more active physically (0.02 vs. − 0.04, *p* < .01) than excluded participants. There was no attrition bias in body mass index or in the use of antihypertensive medications. Regarding socioeconomic factors, included participants had more educational years (15.61 vs. 14.85, *p* < .001) than excluded participants, but there was no attrition bias in the level of income. Finally, included participants’ parents had slightly higher level of income (4.91 vs. 4.73, *p* < .01) and were less likely to have low educational level (32.1% vs. 36.2%, *p* < .05) than excluded participants’ parents.

**Table 1:** The means, standard deviations (SD), frequencies, and ranges of the study variables

Variable (measurement year)	Mean	SD	Range	Frequency (%)
Age in 2001 (years)	31.67	5.03	24; 39	
Sex (female)				757 (58.1)
Parents				
Hypertension				818 (63.2)
Educational level (1980)				
Comprehensive school				418 (32.1)
High school or occupational school				539 (41.4)
Academic level				346 (26.6)
Level of income (1980)	4.91	1.92	1; 8	
Participants				
Compassion in 2001^1^	3.68	0.64	1; 5	
Systolic blood pressure (mmHg)				
2001	115.96	12.90	80.67; 166.67	
2007	120.04	13.90	77.33; 168.67	
2011	118.26	13.90	83.33; 178.67	
Diastolic blood pressure (mmHg)				
2001	70.33	10.35	40.00; 111.33	
2007	75.23	11.08	42.00; 120.00	
2011	74.44	10.24	42.00; 113.33	
Use of antihypertensive medications				
2001				26 (2.0)
2007				82 (6.8)
2011				122 (9.5)
Hypertension (2007/2011)				115 (8.8)
Coffee consumption (cups per day) (2001)	3.25	2.60	0; 18	
Daily smoker (2007/2011)				229 (17.6)
Alcohol use (2007/2011)	4.59	1.24	1; 6	
Physical activity (2007/2011)	0.02	0.64	− 1.66; 1.86	
Body mass index (2007/2011)	25.69	4.47	16.86; 54.47	
Number of educational years (2011)	15.61	3.58	8; 34	
Level of income (2011)	7.32	3.02	1; 13	

^1^Unstandardized value of compassion. In the analyses, the standardized value of compassion (mean = 0, SD = 1) were used. The descriptive statistics were calculated for all the participants who were included in any analysis of this study (i.e., analysis about blood pressure or analysis about hypertension). Moreover, for alcohol use, physical activity, and body mass index, the mean between the measurement years was used

**Fig. 1:**
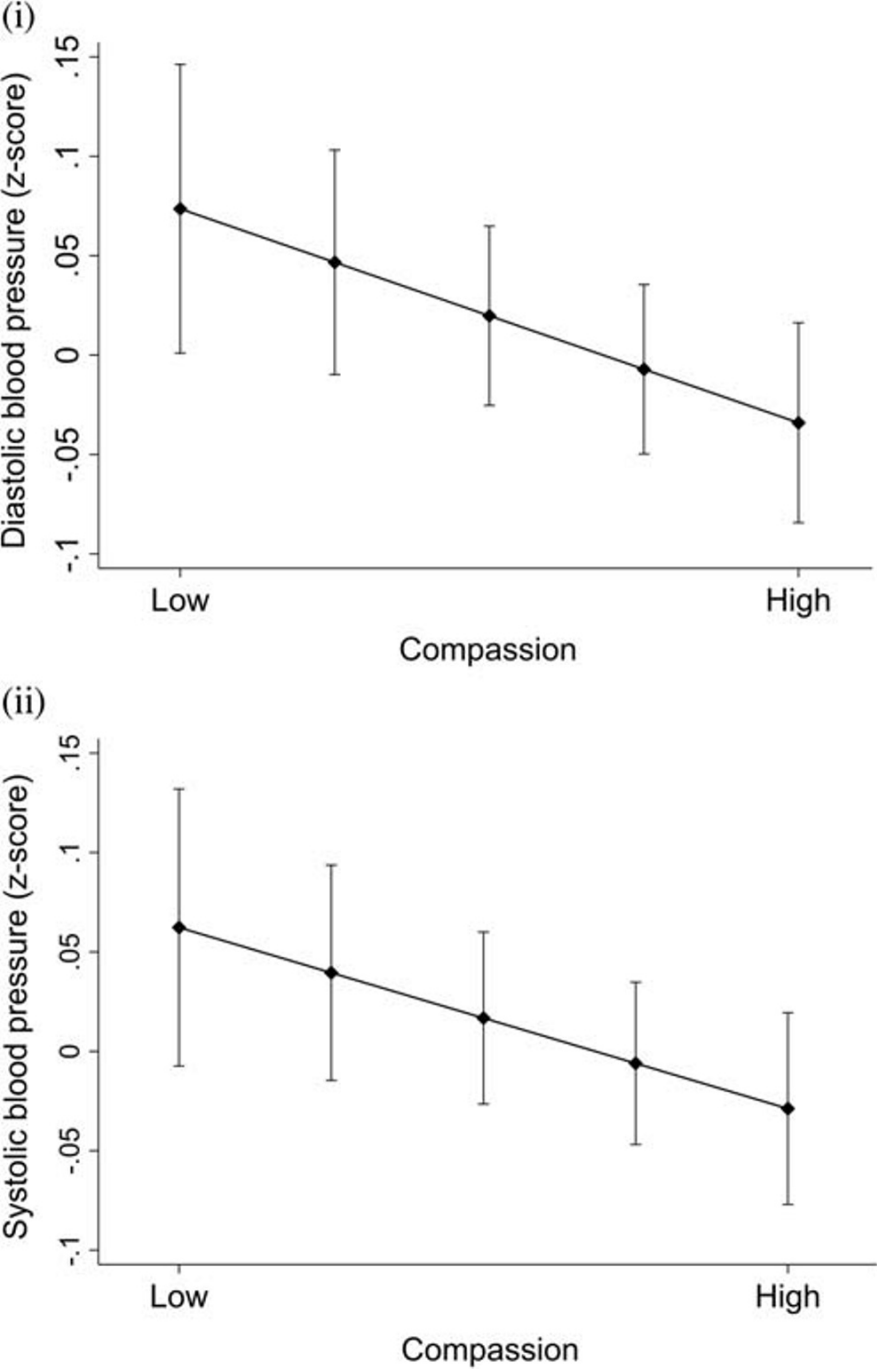
Predicted means with 95% confidence intervals of (i) diastolic blood pressure and (ii) systolic blood pressure (measured in 2007/2011) with different levels of compassion (measured in 2001), ranging from low (10th percentile) to high (90th percentile). Adjusted for age, sex, the use of antihypertensive medications in 2001, 2007, and 2011, and the baseline level of diastolic/systolic blood pressure in 2001

Table 2 shows the results of linear regression analyses when predicting diastolic and systolic blood pressure in 2007–2011 by compassion in 2001. The interaction effect of compassion with familial risk for hypertension was non-significant when predicting diastolic blood pressure (*p* = .330) and systolic blood pressure (*p* = .636). Hence, familial risk for hypertension was excluded from the models. Subsequently, the results revealed that high compassion predicted significantly lower levels of diastolic and systolic blood pressure when adjusted for age, sex, and parents’ and offspring’s socioeconomic factors (see Fig. 1). When adjusted also for participants’ health behaviors, the effect of compassion on diastolic and systolic blood pressure became non-significant.

**Table 2:** The results of linear regression analyses when predicting blood pressure by compassion. Estimates with 95% confidence intervals (CI)

	Model 1			Model 2			Model 3		
	*B*	95% CI	Beta	*B*	95% CI	Beta	*B*	95% CI	Beta
Diastolic blood pressure (2007/2011)									
Age	0.13**	0.05; 0.22	0.07	0.08	− 0.01; 0.17	0.04	0.06	− 0.03; 0.15	0.03
Sex^1^	3.13***	2.28; 3.99	0.16	3.22***	2.33; 4.12	0.16	2.77***	1.82; 3.72	0.14
Baseline diastolic blood pressure (2001)	0.58***	0.54; 0.63	0.61	0.58***	0.54; 0.63	0.62	0.55***	0.51; 0.60	0.59
Parents’ level of income (1980)				− 0.10	− 0.59; 0.38	− 0.01	0.01	− 0.46; 0.49	0.00
Parents’ educational level (1980)				− 0.82*	− 1.48; − 0.17	− 0.06	− 0.86*	− 1.51; − 0.21	− 0.07
Participants’ level of income (2011)				− 0.10	− 0.59; 0.38	− 0.02	− 0.23	− 0.68; 0.23	− 0.02
Participants’ years of education (2011)				− 0.65**	− 1.09; − 0.21	− 0.07	− 0.55*	− 0.99; − 0.11	− 0.06
Coffee consumption (2001)							0.12	− 0.34; 0.59	0.01
Smoking status^2^ (2007/2011)							− 0.80	− 1.97; 0.37	− 0.03
Alcohol use (2007/2011)							− 0.42	− 0.92; 0.08	− 0.04
Body mass index (2007/2011)							1.29***	0.84; 1.75	0.13
Physical activity (2007/2011)							− 0.11	− 0.56; 0.34	− 0.01
Compassion (2001)	− 0.55*	− 0.99; − 0.11	− 0.05	− 0.50*	− 0.94; − 0.07	− 0.05	− 0.45*	− 0.88; − 0.02	− 0.04
Systolic blood pressure (2007/2011)									
Age	0.32***	0.21; 0.43	0.13	0.27***	0.16; 0.38	0.10	0.23***	0.12; 0.35	0.09
Sex^1^	3.15***	2.01; 4.29	0.12	3.10***	1.90; 4.30	0.12	2.48***	1.21; 3.75	0.10
Baseline systolic blood pressure (2001)	0.64***	0.59; 0.68	0.64	0.63***	0.59; 0.68	0.63	0.61***	0.57; 0.66	0.61
Parents’ level of income (1980)				− 0.37	− 0.98; 0.25	− 0.03	− 0.24	− 0.85; 0.37	− 0.02
Parents’ educational level (1980)				− 0.57	− 1.41; 0.26	− 0.03	− 0.60	− 1.43; 0.23	− 0.04
Participants’ level of income (2011)				− 0.03	− 0.60; 0.55	0.00	0.00	− 0.58; 0.58	0.00
Participants’ years of education (2011)				− 1.01***	− 1.57; − 0.45	− 0.08	− 0.84**	− 1.41; − 0.27	− 0.07
Coffee consumption (2001)							0.39	− 0.20; 0.98	0.03
Smoking status^2^ (2007/2011)							− 0.53	− 2.02; 0.96	− 0.02
Alcohol use (2007/2011)							− 0.60	− 1.24; 0.04	− 0.04
Body mass index (2007/2011)							1.19***	0.61; 1.77	0.09
Physical activity (2007/2011)							− 0.30	− 0.88; 0.27	− 0.02
Compassion (2001)	− 0.61*	− 1.17; − 0.05	− 0.04	− 0.56*	− 1.11; − 0.00	− 0.04	− 0.48	− 1.03; 0.07	− 0.04

***p < .001; **p < .01; *p < .05. ^1^Female as the reference group. ^2^Participants without daily smoking as the reference group. *N* = 1112. Model 1, adjusted for age, sex, the baseline level of diastolic/systolic blood pressure, and the use of antihypertensive medications. Model 2, adjusted also for parents’ and offspring’s socioeconomic factors. Model 3, adjusted also for health behaviors. Compassion was standardized to mean = 0 and SD = 1. Hence, *B* refers to change in blood pressure (mmHg) per one-unit change (i.e., 1-SD change) in compassion

When predicting offspring’s hypertension by compassion in the total sample (without considering familial risk for hypertension), compassion did not have significant effect on hypertension in any of the models (*p* > .05) (for further information, see Supplementary Table 2). After adding familial risk for hypertension and the interaction effect between compassion and familial risk for hypertension, there was a significant positive interaction effect between compassion and the familial risk for hypertension (*p* = .030) in predicting offspring’s hypertension.

**Fig. 2:**
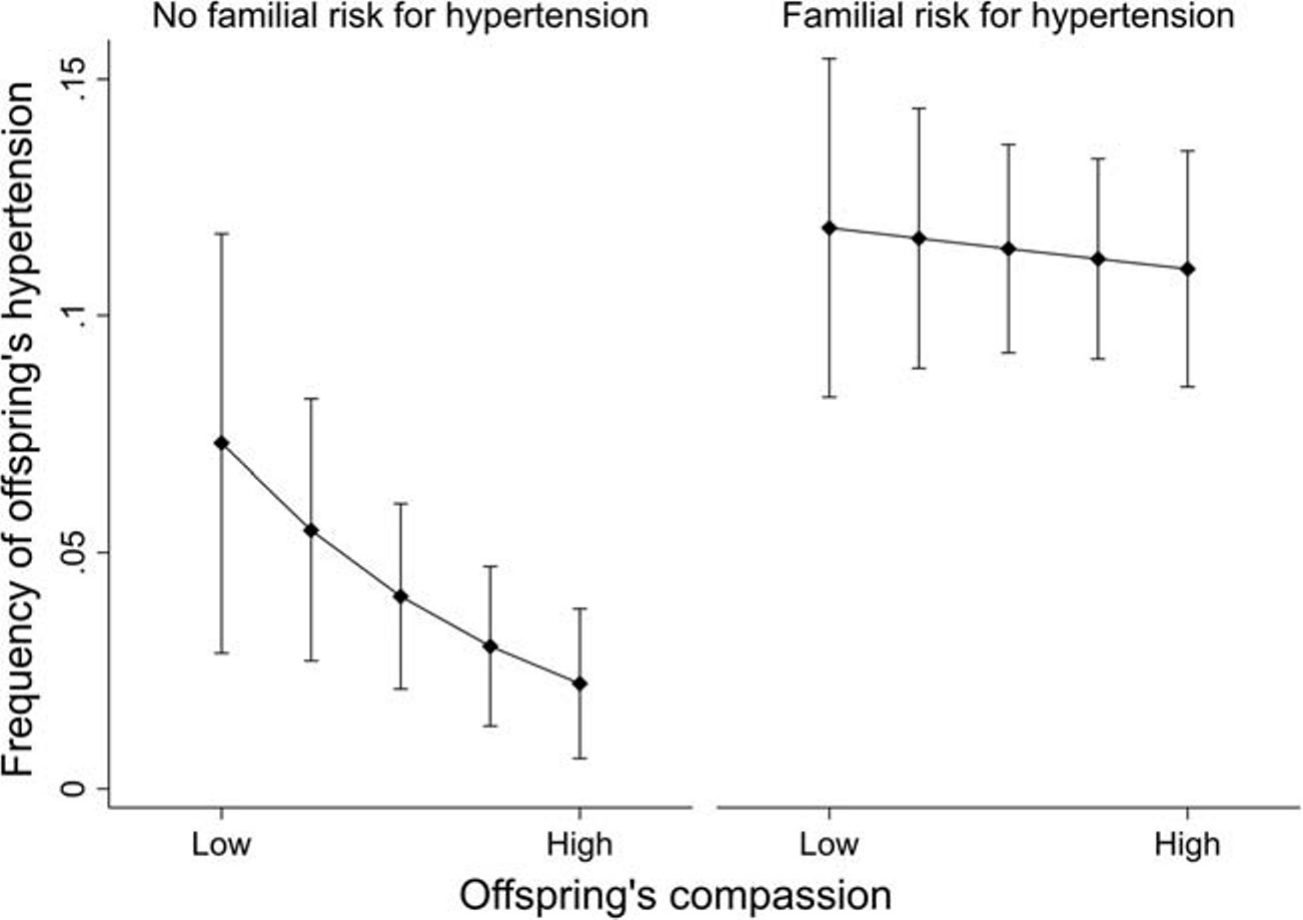
Predicted frequencies (%) with 95% confidence intervals of offspring’s hypertension (measured in 2007/2011) separately for participants with/without familial risk for hypertension (measured in 1980–2007) and with different levels of compassion (measured in 2001), ranging from low (10th percentile) to high (90th percentile). Adjusted for age and sex

Because there was a significant interaction effect between familial risk for hypertension and compassion, the association of offspring’s compassion with hypertension was investigated separately among participants with and without familial risk for hypertension. Among individuals without familial risk for hypertension (Table 3), a 1-SD increase in compassion was related to 0.49 times lower odds for hypertension. Instead, among individuals with high familial risk for hypertension (Table 3), compassion was not related to hypertension. The effects were independent of age, sex, offspring’s and their parents’ socioeconomic factors, and health behaviors (i.e., smoking status, alcohol use, body mass index, physical activity, and coffee consumption). These results are illustrated in Fig. 2 where high compassion predicted a lower risk for hypertension among individuals without familial risk for hypertension but not among individuals with familial risk for hypertension.

**Table 3:** The results of logistic regression analyses when predicting hypertension in 2007/2011 by compassion among participants with (*N* = 817) and without (*N* = 476) familial risk for hypertension. Estimates (*B*) and odds ratios (OR) with 95% confidence intervals (CI)

	Model 1			Model 2			Model 3		
	*B*	OR	95% CI	*B*	OR	95% CI	*B*	OR	95% CI
Among participants without familial risk for hypertension									
Age	0.18**	1.20	1.07; 1.36	0.18**	1.20	1.06; 1.36	0.20**	1.22	1.06; 1.40
Sex^1^	1.03	2.80	0.92; 8.50	0.87	2.38	0.70; 8.09	0.79	2.21	0.52; 9.37
Parents’ level of income (1980)				− 0.36	0.70	0.40; 1.22	− 0.29	0.75	0.40; 1.40
Parents’ educational level (1980)				− 0.14	0.87	0.37; 2.08	− 0.18	0.85	0.31; 2.25
Participants’ level of income (2011)				0.39	1.48	0.81; 2.68	0.54	1.71	0.87; 3.36
Participants’ years of education (2011)				− 0.10	0.91	0.51; 1.61	− 0.17	0.85	0.42; 1.69
Coffee consumption (2001)							− 0.09	0.91	0.47; 1.75
Smoking status^2^ (2007/2011)							− 0.78	0.46	0.07; 2.83
Alcohol use (2007/2011)							0.00	1.00	0.51; 1.98
Body mass index (2007/2011)							1.05***	2.85	1.70; 4.77
Physical activity (2007/2011)							− 0.28	0.76	0.43; 1.33
Compassion (2001)	− 0.66*	0.52	0.30; 0.89	− 0.69*	0.50*	0.29; 0.87	− 0.71*	0.49	0.26; 0.93
Among participants with familial risk for hypertension									
Age	0.12***	1.13	1.07; 1.18	0.12***	1.12	1.07; 1.18	0.10***	1.10	1.04; 1.17
Sex^1^	0.31	1.36	0.88; 2.11	0.46	1.59	1.00; 2.53	0.15	1.16	0.70; 2.01
Parents’ level of income (1980)				0.06	1.06	0.81; 1.38	0.16	1.18	0.88; 1.57
Parents’ educational level (1980)				− 0.12	0.89	0.62; 1.26	− 0.19	0.83	0.57; 1.21
Participants’ level of income (2011)				− 0.29	0.75*	0.58; 0.96	− 0.29*	0.75	0.57; 0.98
Participants’ years of education (2011)				− 0.04	0.96	0.75; 1.23	− 0.02	0.98	0.76; 1.27
Coffee consumption (2001)							0.00	1.00	0.78; 1.28
Smoking status^2^ (2007/2011)							− 0.66	0.52	0.26; 1.04
Alcohol use (2007/2011)							− 0.37**	0.69	0.53; 0.90
Body mass index (2007/2011)							0.79***	2.21	1.76; 2.77
Physical activity (2007/2011)							− 0.22	0.80	0.62; 1.04
Compassion (2001)	− 0.05	0.95	0.76; 1.20	− 0.04	0.96	0.77; 1.21	0.03	1.03	0.80; 1.33

***p < .001; **p < .01; *p < .05. ^1^Female as the reference group. ^2^Participants without daily smoking as the reference group. Total *N* = 1293. Model 1, adjusted for age and sex. Model 2, adjusted also for parents’ and offspring’s socioeconomic factors. Model 3, adjusted also for health behaviors. Compassion was standardized to mean = 0 and SD = 1. Hence, OR refers to change in probability of hypertension per one-unit change (i.e., 1-SD change) in compassion

## Discussion

This was the first study to investigate the relationship of compassion for others with diastolic and systolic blood pressure and hypertension and whether familial risk might modify this relationship. High compassion predicted slightly lower levels of diastolic and systolic blood pressure in adulthood both among individuals with and without familial risk for hypertension. Additionally, familial risk for hypertension modified the association of compassion with hypertension in adulthood. That is, among individuals with no familial risk for hypertension, high compassion for others predicted a decreased risk for hypertension in adulthood, whereas among individuals with familial risk for hypertension, compassion for others was not related to hypertension in adulthood. This suggests that compassion has a health-protective effect but only in individuals who are at low genetic risk for hypertension.

We found that the effect of compassion on blood pressure somewhat attenuated after controlling for health behaviors, implying that the relationship of compassion with blood pressure may partially proceed via favorable health behaviors. This is in line with previous studies demonstrating that compassion is related to a lower risk for excessive energy intake, smoking, substance use, and caffeine-including drinks [19–22, 40] that, in turn, are linked with lower blood pressure [28, 41, 42]. Importantly, it has been suggested that certain types of health behaviors may have only a relatively transient effect on blood pressure over time. For example, alcohol use, physical activity, and coffee consumption may predict changes in blood pressure over the following hours but not thereafter [43–46]. Correspondingly, interventions targeting health behaviors are shown to effectively lower the level of blood pressure comparatively rapidly [47–49]. This may potentially provide one explanation why the link of compassion with variations in blood pressure levels (at single measurement times) may partially proceed via health behaviors, whereas the link of compassion with hypertension (i.e., a more stable and long-lasting form of raised blood pressure) was not attenuated and actually rather became stronger after controlling for health behaviors health behaviors.

Hence, our findings suggested that the association of high compassion with a decreased risk for hypertension may proceed via some other pathways than health behaviors. Generally, it has been suggested that psychological factors may protect against raised blood pressure by altering central nervous system control of such physiological reactivity patterns that are related to cardiovascular functioning [18, 50]. By now, there is evidence that compassionate states are related to lower heart rate, higher respiratory sinus arrhythmia, and higher heart rate variability [51, 52] that, in turn, are linked with a lower risk for hypertension [53, 54]. Moreover, brain imaging studies have suggested that high compassion is related to higher activity in such brain structures that contribute to the regulation of blood pressure [55]. Taken together, hypertension is a comparatively stable trait resulting from a complex pattern of cardiovascular, endocrinological, and respiratory functioning [56], and this pattern may be affected by compassion.

Previously, several studies have found that individuals with genetic risk factors are more susceptible for environmental risk factors, when predicting coronary heart disease and myocardial infarction [57, 58]. Consequently, environmental risk factors and genetic vulnerabilities are suggested to be cumulated in the pathology of cardiovascular diseases [59]. This may result from epigenetic effects, indicating that environmental stressors activate heart disease–related genes that, in turn, predispose to increased risk for coronary heart disease or infarction [60].

Importantly, however, it has been highlighted that empirical evidence for the interplay between genetic and environmental factors in the context of hypertension is very limited [61]. Our findings suggested that high compassion predicts a decreased risk for hypertension only among individuals without familial risk for hypertension. This is in line with the statements that among individuals with a lack of genetic risk for hypertension, the protective role of psychological factors may be significant [28]. That is, in case an individual has lower genetic risk for hypertension, there may exist more variance in the incidence of hypertension to be explained by psychosocial factors such as compassion. This is also in line with a previous study reporting that amount of exercise had no effect on blood pressure in individuals with a specific genetic variant [62]. Moreover, as the lack of familial risk for hypertension may likely imply the presence of protective genes against hypertension, our results suggest that the protective effect of compassion may cumulate with protective genetic factors in the development and maintenance of healthy blood pressure levels.

Previously, it has been found that the role of genetic factors in the etiology of hypertension is especially strong in early-onset hypertension (i.e., onset before 55 years age) [63]. In the present study, the participants were aged below 55 years (34–49 years), so that all the cases of offspring’s hypertension could be classified as early-onset hypertension. Future studies could investigate whether the protective role of compassion might be stronger against late-onset hypertension that is less strongly transmitted from one generation to the next than early-onset hypertension [26], implying that the incidence of late-onset hypertension might be especially susceptible to psychosocial factors.

The present study had some methodological limitations that must be taken into consideration. Firstly, data were not available on participants’ hypertension in 2001. Hence, no firm conclusions can be made whether the hypertensive cases were incident before or after the measurement of compassion. However, because the youngest participants were only 24-year-olds at the measurement time of compassion, it is likely that most of the hypertension cases have emerged thereafter. Secondly, hypertension was assessed with self-report questionnaires, instead of using data from health care registers. Nevertheless, there is evidence that the self-reports of hypertension show an acceptable agreement with information from medical databases [64] and provide a highly accurate estimate of presence of hypertension [65, 66]. Additionally, parents were asked whether they had been diagnosed with hypertension by a doctor, not whether they mere thought having hypertension. Moreover, it has been demonstrated that the prevalence of undiagnosed hypertension is comparatively low among the Finnish population [67], suggesting that the knowledge of hypertension is at a high level in Finland. Hence, it has been concluded that self-reports can be used, with caution, as a measure of raised blood pressure or hypertension [64, 65].

Thirdly, compassion was measured with a self-rating questionnaire; it was susceptible to social desirability bias. Nevertheless, it is challenging to measure compassion with other measures than self-ratings since it reflects one’s internal feelings and experiences. Compassionate states could be measured with, for example, some electrophysiological measures such as heart rate variability [51]. However, heart rate variability does not reflect experience of compassion on a quite stable level over situations, and therefore was not suitable for the present study. Moreover, the reliability and validity of the compassion scale are shown to be high [31, 33, 34] and self-rating methods have been used also previously [52].

The present study had also several substantial strengths. Firstly, to our knowledge, this was the first study to investigate the relationship of compassion for others with blood pressure and hypertension. Secondly, this study used a comparatively large population-based sample (*N* = 1112–1293) that was likely to present the general population with regard to most of its characteristics. Thirdly, this study used intergenerational data with a 31-year prospective follow-up that enabled us to investigate whether familial risk for hypertension modifies the association of compassion with raised blood pressure. This kind of prospective study, clarifying how psychological and biological factors interact in the etiology of hypertension, has previously been demanded (5) but has not been conducted previously. Fourthly, this study could clarify potential pathways from compassion to blood pressure and hypertension by controlling for a variety of factors, such as age, sex, use of antihypertensive medications, socioeconomic factors, and health behaviors (coffee consumption, alcohol use, smoking status, body mass index, physical activity).

Previously, it has been estimated that the worldwide prevalence of hypertension is about 31% [68]. Among individuals with raised blood pressure, about 77% are using antihypertensive medications [69]. However, a severe concern has been expressed that antihypertensive medications seem not to be effective for a great portion of hypertensive patients [28], with estimates ranging from 12% to even 40% of the drug-treated population [70–72]. Furthermore, a substantial number of hypertensive patients experience a wide variety of side effect symptoms related to antihypertensive medications, for example, fatigue, insomnia, depressive symptoms, and weight gain [73].

Hence, there has been a wide consensus that, besides antihypertensive medications and lifestyle changes, hypertensive patients should be provided also with psychosocial interventions that aim at increasing stress management and relaxation [74–76]. Importantly, however, several intervention studies and meta-analyses have concluded that the current relaxation techniques, stress reduction programs, or cognitive-behavioral therapies seem not to be effective treatment methods for raised blood pressure [77–79]. Hence, there is an acute need for the development of novel psychosocial treatments. By now, intervention studies have suggested that practicing compassion might be a comparatively effective method to alleviate a range of psychiatric symptoms, such as depression, anxiety, stress, and psychotic symptoms [80, 81]. Our findings suggest that high compassion may predict lower diastolic and systolic blood pressure and also protect against hypertension among individuals without familial risk for hypertension. Future studies could investigate whether compassion-enhancing interventions might have beneficial effects on blood pressure.

## Electronic Supplementary Material

ESM 1 (DOCX 40 kb).
