# Supplementary material for: Does Compassion Predict Blood Pressure and Hypertension? The Modifying Role of Familial Risk for Hypertension
Source: Int J Behav Med. 2020 Apr 28;27(5):527–38. doi: 10.1007/s12529-020-09886-5 (PMC7497423; doi:10.1007/s12529-020-09886-5)
Supplement: Supplementary file 1 — (DOCX 40 kb). [file 12529_2020_9886_MOESM1_ESM.docx]

**Supplementary Material.** The items of the scale of physical activity.

1. “How much breathlessness and sweating do you experience when you engage in sport or physical activity?” (1=not at all; 3=a lot)
2. “How often do you engage in sport or physical activity so that you get out of breath and sweat?” (1=never; 6=daily)
3. “How many hours per week do you usually engage in sport or physical activity so that you get out of breath and sweat?” (1=not at all; 6=7 hours or more)
4. “How much time do you usually spend in one session of sport of physical activity?” (1=less than 20 minutes; 4=more than 60 minutes)
5. “Do you participate in organized physical activity (e.g. in sport club)?” (1=not at all; 4=several hours per week).

**Supplementary Table 1.** The timeline of the study design.

|  | 1980 | 1983 | 1986 | 1989 | 1992 | 2001 | 2007 | 2011 |
| --- | --- | --- | --- | --- | --- | --- | --- | --- |
| Generation I: Parents |  |  |  |  |  |  |  |  |
| Socioeconomic factors | X |  |  |  |  |  |  |  |
| Hypertension | X | X | X | X | X | X | X |  |
| Generation II: Offspring |  |  |  |  |  |  |  |  |
| Blood pressure |  |  |  |  |  | X | X | X |
| Anti-hypertensive medications |  |  |  |  |  | X | X | X |
| Hypertension |  |  |  |  |  |  | X | X |
| Health behavior |  |  |  |  |  | X | X | X |
| Coffee consumption |  |  |  |  |  | X |  |  |
| Socioeconomic factors |  |  |  |  |  |  |  | X |
| Compassion |  |  |  |  |  | X |  |  |

**Supplementary Table 2.** The results of logistic regression analyses when predicting offspring’s hypertension by compassion hypertension in the total sample. Coefficients (B) and odds ratios (OR) with 95% confidence intervals (CI).

|  | Hypertension | | | | | | | | | | |
| --- | --- | --- | --- | --- | --- | --- | --- | --- | --- | --- | --- |
|  | Model 1 | | |  | Model 2 | | |  | Model 3 | | |
|  | B | OR | 95% CI |  | B | OR | 95% CI |  | B | OR | 95% CI |
| Age | 0.14*** | 1.15 | 1.10; 1.20 |  | 0.13*** | 1.14 | 1.09; 1.20 |  | 0.12*** | 1.12 | 1.07; 1.18 |
| Sex^1^ | 0.42* | 1.52 | 1.02; 2.26 |  | 0.51* | 1.67 | 1.09; 2.55 |  | 0.21 | 1.24 | 0.76; 2.03 |
| Parents' level of income |  |  |  |  | 0.00 | 1.00 | 0.79; 1.25 |  | 0.10 | 1.10 | 0.86; 1.41 |
| Parent's educational level |  |  |  |  | -0.11 | 0.89 | 0.65; 1.24 |  | -0.19 | 0.82 | 0.58; 1.17 |
| Participants' level of income |  |  |  |  | -0.19 | 0.83 | 0.66; 1.03 |  | -0.17 | 0.84 | 0.66; 1.07 |
| Participants' years of education |  |  |  |  | -0.07 | 0.93 | -0.75; 1.16 |  | -0.04 | 0.96 | 0.76; 1.22 |
| Coffee consumption |  |  |  |  |  |  |  |  | 0.01 | 1.01 | 0.80; 1.27 |
| Smoking status^2^ |  |  |  |  |  |  |  |  | -0.57 | 0.57 | 0.30; 1.07 |
| Alcohol use |  |  |  |  |  |  |  |  | -0.37** | 0.69 | 0.54; 0.88 |
| Body-mass index |  |  |  |  |  |  |  |  | 0.82*** | 2.28 | 1.86; 2.78 |
| Physical activity |  |  |  |  |  |  |  |  | -0.23 | .079 | 0.63; 1.00 |
| Compassion^3^ | -0.17 | 0.84 | 0.69; 1.03 |  | -0.16 | 0.85 | 0.69; 1.05 |  | -0.10 | 0.90 | 0.72; 1.13 |
| *** *p*<.001 ** *p*<.01 * *p*<.05 ^1^ Female as the reference group. ^2^ Participants without daily smoking as the reference group. ^3^ Standardized with the mean of 0 and SD of 1. *N*=1293 | | | | | | | | | | | |
| Model 1: Adjusted for age and sex. | | | | | | | | | | | |
| Model 2: Adjusted also for parents' and offspring's socioeconomic factors. | | | | | | | | | | | |
| Model 3: Adjusted also for coffee consumption, smoking status, alcohol use, body-mass index, and physical activity. | | | | | | | | | | | |
| *Note*: Compassion was standardized to mean=0 and SD=1. Hence, OR refers to change in probability of hypertension per one-unit change (i.e. 1-SD change) in compassion. | | | | | | | | | | | |
